# Altered Local Gyrification Index and Corresponding Functional Connectivity in Medication Free Major Depressive Disorder

**DOI:** 10.3389/fpsyt.2020.585401

**Published:** 2020-12-14

**Authors:** Jiang Long, Jinping Xu, Xue Wang, Jin Li, Shan Rao, Huawang Wu, Weihong Kuang

**Affiliations:** ^1^Deparment of Psychiatry, West China Hospital, Sichuan University, Chengdu, China; ^2^Institute of Biomedical and Health Engineering, Shenzhen Institutes of Advanced Technology, Chinese Academy of Sciences, Shenzhen, China; ^3^Department of Radiology, The Affiliated Brain Hospital of Guangzhou Medical University (Guangzhou Huiai Hospital), Guangzhou, China; ^4^Department of Psychiatry and National Clinical Research Center for Geriatrics, West China Hospital, Sichuan University, Chengdu, China

**Keywords:** major depressive disorder, resting-state, functional connectivity, fMRI, local gyrification index

## Abstract

A lot of previous studies have documented that major depressive disorder (MDD) is a developmental disorder. The cortical surface measure, local gyrification index (LGI), can well reflect the fetal and early postnatal neurodevelopmental processes. Thus, LGI may provide new insight for the neuropathology of MDD. The previous studies only focused on the surface structural abnormality, but how the structural abnormality lead to functional connectivity changes is unexplored. In this study, we investigated LGI and corresponding functional connectivity difference in 28 medication-free MDD patients. We found significantly decreased LGI in left lingual gyrus (LING) and right posterior superior temporal sulcus (bSTS), and the changed LGI in bSTS was negatively correlated with disease onset age and anxiety scores. The following functional connectivity analyses identified decreased functional connectivities between LING and right LING, precentral gyrus, and middle temporal gyrus. The decreased functional connectivities were correlated with disease duration, onset, and depression symptoms. Our findings revealed abnormal LGI in LING and bSTS indicating that the abnormal developmental of visual and social cognition related brain areas may be an early biomarker for depression.

## Introduction

Major depressive disorder (MDD) is one of the most common psychiatric disorder and the leading causes of ill health and disability worldwide (1, 2). Although a large number of studies have revealed structural and functional abnormalities in MDD patients (3–12), the underlaying neural basis of MDD is still unclear. Emerging evidence has demonstrated that neurodevelopmental variation was related to the increased lifetime risk for the onset of MDD suggesting variations of cortical development may contribute largely to the neuropathology of MDD (13–15). Therefore, characterization of the cortical developmental patterns may provide a new way to reveal the neuronal basis of MDD.

The cortical structural changes in MDD are usually studied with voxel-based morphometry approach to delineate the cortical gray matter volume. Given that gray matter volume includes the information of cortical thickness, surface area, and cortical folding (16), thus, to direct investigation of thickness, surface area, and cortical folding patterns which reflect different biological factors is better and more accurate to reveal the structural alterations in MDD (17, 18). The cortical thickness and surface area mainly characterize the neuronal density and the number and spacing of cortical columns, respectively (19–21). The cortical folding defined by local gyrification index (LGI) is considered to be related to the fetal and early postnatal neurodevelopmental processes (22–24). Thus, LGI is the most ideal method to investigate the abnormal neurodevelopmental patterns in MDD. With LGI method, some studies have found the changed LGI in MDD compared to healthy controls (25–27). Schmitgen et al. (27) found significantly greater LGI in frontal, cingulate, parietal, temporal, and occipital regions in MDD patients. Han et al. (26) found increased LGI in rostral anterior cingulate cortex, medial orbitofrontal cortex and frontal pole in MDD patients using region of interest analysis. In addition, Zhang et al. found decreased LGI in middle cingulate cortex, insula, orbital frontal cortex, anterior cingulate cortex and temporal operculum (25). Although these studies found abnormal LGI in MDD, the studies of Schmitgen et al. (27) and Han et al. (26) included the patients with medication and the study of Zhang et al. (25) only included a small sample drug-naïve MDD patients (18 patients). Moreover, the previou findigns in different studies varied greatly, which needs to be further validated. In addition, the previous studies only revealed the LGI changes, but the associated changes of functional couplings are unexplored.

In this study, we applied the LGI method to study the abnormal development effects on brain structure and corresponding functional connectivities in 28 medication-free MDD patients and 30 gender-, age-, education-level matched healthy controls.

## Materials and Methods

### Participants

Twenty-eight medication-free in the current episode MDD patients were recruited from the Affiliated Brain Hospital of Guangzhou Medical University and diagnosed with the Diagnostic and Statistical Manual of Mental Disorders-IV criteria. Thirty gender-, age-, and educational level matched healthy controls (HC) were also recruited. All participants were right-handed, and the severities of depressive and anxious symptoms were rated using 24-item Hamilton Rating Scale for Depression (HRSD) (28) and Hamilton Anxiety Rating Scale (HAMA) (29), respectively. In this study, the subjects who were left-handed, substance dependence, pregnant, life threatening somatic disease, neurological disorders, other comorbid mental disorders or MRI-related contraindications were excluded. All the subjects provided written informed consent. The study was in accordance with the latest revision of the declaration of Helsinki and fully approved by the Affiliated Brain Hospital of Guangzhou Medical University Ethics Committee. The demographic and psychological characteristics of the samples are listed in Table 1.

**Table 1:** Demographics and clinical characteristics of the subjects used in present study.

	**MDD (*n* = 30)**	**HC (*n* = 28)**	***P* value**
Age (years)	33.43 ± 11.76	32.00 ±9.13	0.61
Gender (male/female)	10/20	10/18	0.85
Education level	13.75 ± 4.05	13.5± 3.04	0.79
Onset age (years)	31.90 ± 12.23		
Duration of illness (weeks)	92.66 ± 122.81		
HRSD scores	31.2 ± 6.90		
HAMA scores	19.48 ± 7.08		

*A Pearson chi-squared test was used for gender comparison. Two-sample t-tests were used for age, education comparisons. HRSD, Hamilton Depression Rating Scale score; HAMA, Hamilton Anxiety Scale score; MDD, major depression disorder; HC, healthy control*.

### MRI Data Acquisition

All the subjects MRI data were scanned using a 3.0-Tesla Philips MR imaging system from the Affiliated Brain Hospital of Guangzhou Medical University. They were instructed to keep their eyes closed, be relaxed, awake, and not to think of anything during the scan. The T1-weighted anatomic images were acquired with the following parameters: repetition time (TR) = 8.2 ms, echo time (TE) = 3.7 ms, flip angle (FA) = 7°; field of view (FOV) = 256 × 256 mm^2^; acquisition matrix = 256 × 256; voxels size = 1 × 1 × 1 mm^3^. Resting-state fMRI data were also collected with parameters: TR/TE = 2000/30 ms, acquisition matrix = 64 × 64, slice thickness = 4 mm with inter-slice gap = 0.6 mm, 33 slices, voxel resolution = 3.44 × 3.44 × 4.6 mm, 240 volumes.

### Resting-State fMRI Preprocessing

All the resting-state fMRI data were preprocessed using DPARSFA software (http://www.restfmri.net/forum/DPARSF). The pre-processing steps included: (1), discarding the first 10 volumes for magnetization equilibrium; (2) slice timing for correcting within-scan acquisition differences between slices; (3) registering all the remaining images to the first volume to correct head motion; (4) spatial normalization to the standard EPI template and resampled to 3 mm^3^ voxels and smoothing with 6 mm Gaussian kernel; (5) detrending was performed to remove the linear drift; (6) Friston 24-parameter model of head motion, white matter, cerebrospinal fluid, and global mean signals were regressed out and filtered with band pass 0.01–0.1 Hz. To exclude the head motion effects, if the subjects with head motion exceeding 3 mm or 3° were excluded, and no subjects were excluded under this criterion. Furthermore, a scrubbing method was conducted to exclude bad images with the mean frame displacement (FD) above 0.5 mm, and one volume before and two volumes after the bad volume were discarded (30).

### Surface LGI Analyses

Cortical LGI analysis were performed on T1 structure image using the the Freesurfer 5.0 toolkit (http://surfer.nmr.mgh.harvard.edu/) with the following steps: first, all the subjects were processed using the fully automated FreeSurfer “recon-all” standard procedure; Then, non-uniform intensity correction, skull stripping, Talairach transforms, normalization and atlas registration, subcortical segmentation, surfaces reconstruction, cortical atlas registration and segmentation were performed. Next, the LGI was calculated by measuring the ratio of local surface area to the outer hull layer that tightly wraps the pial surface (31), which is an indication of sulcal cortex buried in its locality and thus denotes the extent of cortical folding (32). Finally, the LGI of the left and right hemisphere were smoothed with a circularly symmetric Gaussian kernel of 5 mm full width half maximum to provide normal distribution of the results. To identify the LGI differences between MDD and HC, independent two-sample *t*-tests were performed, and the results were corrected for multiple comparisons using Monte Carlo simulation (a pre-cached cluster-wise level of *p* < 0.05, a voxel-wise level of *p* < 0.001).

### Resting-State Functional Connectivity Analyses

After the LGI analyses, the surface areas with changed LGI were taken as seed regions for functional connectivity (FC) analyses which was measured using Pearson correlation coefficients. To calculate functional connectivity analysis, the peak coordinates of the seed regions were first obtained and were transformed to MNI space to create spheres with 6 mm radium. Then, the Pearson correlation coefficients between the mean time series of each seed region and that of each voxel of the whole brain were calculated. Next, all the FCs were converted to Z values to improve normality using Fisher r-to-z transformation. Finally, independent two-sample *t*-tests (FD value as covariant) were performed to identify the functional connectivity differences between MDD and HCs. The significance was determined using a cluster-level Alphasim corrected threshold of *p* < 0.05 (cluster-forming threshold at voxel-level *p* < 0.001, and minimum cluster size = 56).

### Correlation Analyses

To determine the relationship between LGI, resting-state FCs and HRSD, HAMA scores, Pearson's correlation analyses were conducted. The significance was set at a threshold of *p* < 0.05.

## Results

### Clinical Characteristics

There were no significant difference in gender (*p* = 0.85), age (*p* = 0.61), and education levels (*p* = 0.79) between MDD patients and HCs (Table 1).

### Vertex-Wise Differences in LGI

**Figure 1:**
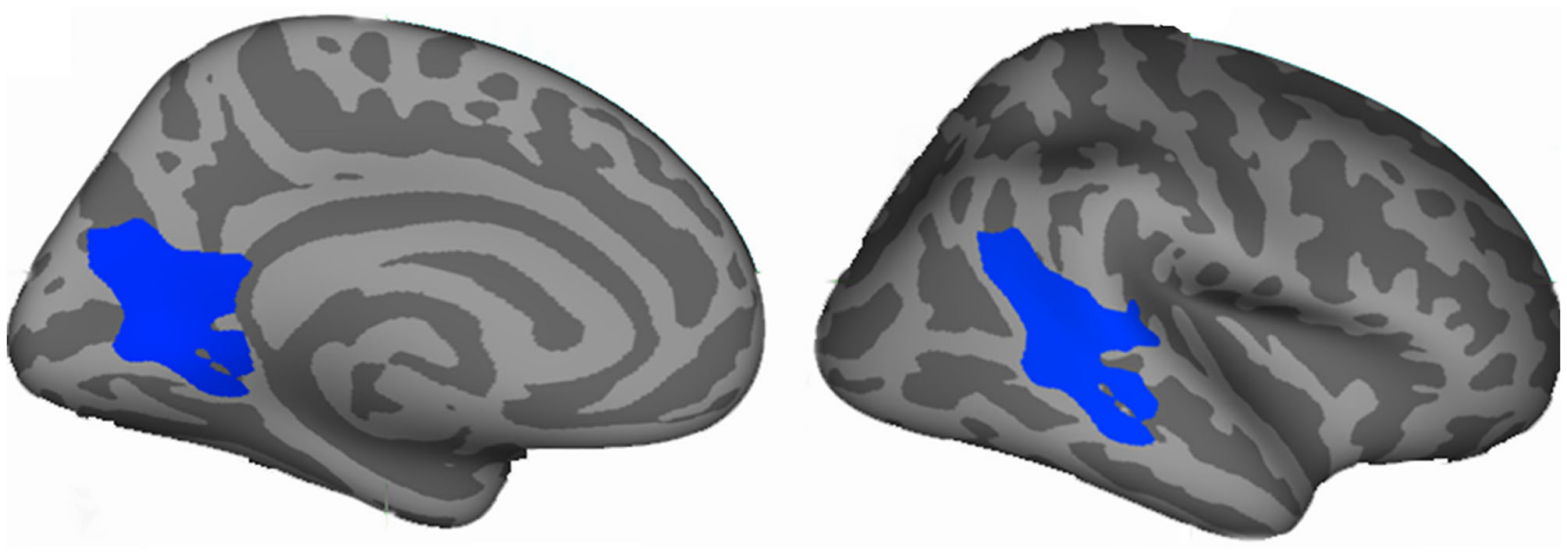
Vertex-wise analyses identified changed cortical local gyri index (LGI) in the major depressive disorder (MDD). Decreased LGI in left lingual gyrus (LING) and right posterior superior temporal sulcus (bSTS) were found in MDD patients compared to healthy controls. The coordinates are MNI space peak locations.

Decreased vertex-wise LGI values were observed in the left lingual gyrus (LING), and right posterior superior temporal sulcus (bSTS) in MDD patients compared to HCs (Figure 1, Table 2).

**Table 2:** Regions with differences in LGI and FC in MDD patients.

**Parameters**	**Brain regions**	**BA**	**Peak MNI coordinates**			***t* value**	**Cluster size**
			**X**	**Y**	**Z**		
LGI:	Lingual gyrus	18	−15	−71	8	−3.81	2,188
	Superior temporal sulcus	21	67	−47	3	−3.53	2,250
FC:	Precentral gyrus	4	25	−29	72	−4.34	98
	Anterior temporal gyrus	21	59	6	−13	−4.21	122
	Lingual gyrus	19	26	−57	−3	−3.89	65

*LGI, local gyrification index; FC, functional connectivity; BA, Brodmann area; MNI, Montreal Neurological Institute; MDD, major depression disorder*.

### Functional Connectivity Analyses

**Figure 2:**
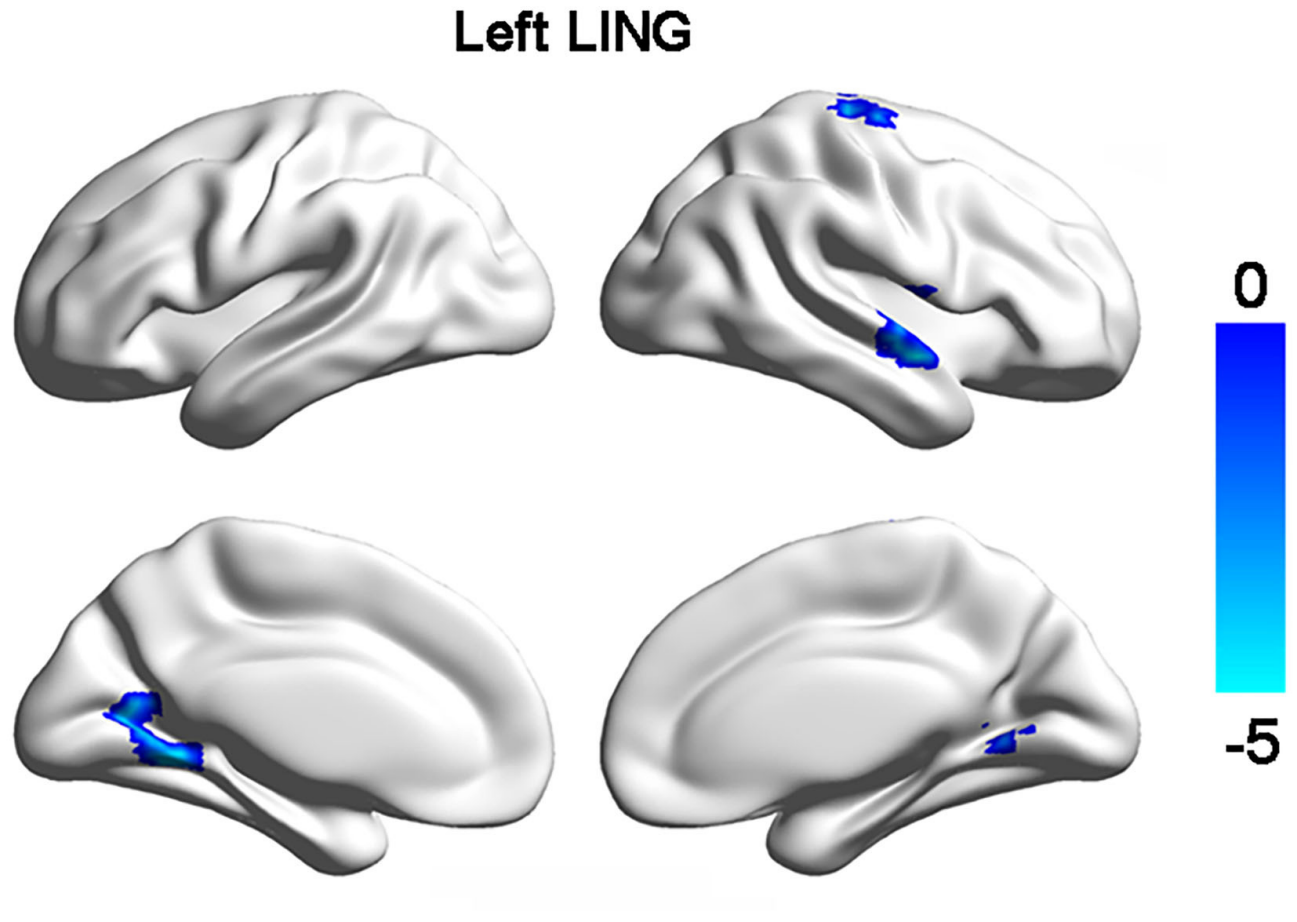
Changed functional connections with left lingual gyrus (LING). Decreased functional connectivities of left LING with right LING, dorsal precentral gyrus, and anterior superior temporal gyrus were found in MDD patients compared to healthy controls.

Resting-state functional connectivity analyses only identified significant decrease of FCs between left lingual gyrus and right dorsal precentral gyrus (PreCG), right anterior temporal gyrus, right lingual gyrus in MDD patients (Figure 2, Table 2). No significant difference in FCs of bSTS was found.

### Correlation Analyses

**Figure 3:**
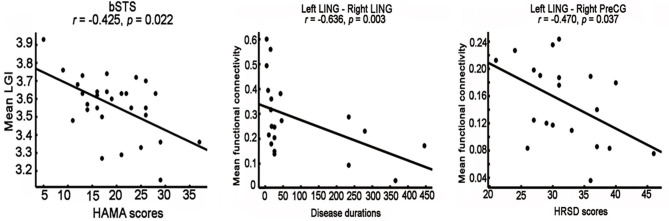
Correlation analyses between neural indices and clinical performances. significantly negative correlations between the mean local gyri index (LGI) of right posterior superior temporal sulcus (bSTS), functional connectivity between left and right lingual gyrus (LING), functional connectivity between left LING and right dorsal precentral gyrus (PreCG) and HAMA scores, disease duration, HRSD scores were identified, respectively.

Correlation analyses identified significantly negative associations between the LGI values of bSTS and HAMA scores (*r* = −0.425, *p* = 0.022) (Figure 3). The FCs between left and right lingual gyrus were negatively correlated disease duration (*r* = −0.636, *p* = 0.003), and the FCs between left lingual gyrus and right dorsal PreCG were negatively correlated HRSD scores (*r* = −0.47, *p* = 0.037) (Figure 3).

## Discussion

In the current study, we found that MDD patients showed decreased LGI in left lingual gyrus, right posterior superior temporal sulcus and decreased functional connectivities between left lingual gyrus and right lingual gyrus, dorsal precentral gyrus, anterior superior temporal gyrus. Moreover, the LGI values in left lingual gyrus, functional connectivity between left and right lingual gyrus, functional connectivity between left lingual gyrus and right dorsal precentral gyrus were negatively corrected with HAMA scores, disease duration, and HRSD scores, respectively. All these findings indicated that LGI and functional connectivity are effective tools to reveal the abnormal developmental effects on brain structure and function in MDD patients.

In this study, the decreased LGI in MDD patients compared to healthy controls was found. There are two prevalent theories, including tension-based theory and convolutional development theory, were proposed to account for cortical folding (33, 34). The tension-based theory considers that the cortical folding is related to the forces driving the extensive wiring of cortico-cortical connections along the cortical surface while convolutional development theory thinks that differential growth rates of cortical layers affect the degree of cortical folding. Thus, LGI is a good method to reflect the early neurodevelopment of brain. The decreased LGI in left lingual gyrus and right anterior superior temporal gyrus in MDD might be induced by disrupted development of early white matter or cortical structures.

We found significantly decreased LGI in the left lingual gyrus and the decreased functional connections of left lingual gyrus with right lingual gyrus, dorsal precentral gyrus, and anterior superior temporal gyrus. The abnormal LGI in lingual gyrus was supported by the study of Schmitgen et al. (27). But in Schmitgen et al. (27) study, they found increased LGI in lingual gyrus in MDD patients. The discrepancy may result from the inhomogeneity of the MDD patients used in the two studies and the medication effects. The MDD patients in our study are medication-free but 25 of 38 patients in Schmitgen et al. (27) study received medication treatment. Compared with Zhang et al. (25) and Han et al. (26) studies, no consistent finding of LGI was found. The inconsistency may be due to the differences in sample size and analytical method. The study of Zhang et al. (25) only included 18 MDD patients, and the study of Han et al. (26) only analyzed the LGI in prefrontal cortex and anterior cingulate cortex using region of interest method. Moreover, the lingual gyrus is an important area of ventral visual processing stream and visual recognition circuit mainly participating in object identification (35) and affect identification (36, 37). The disrupted functional connectivity between visual recognition circuit and subgenual anterior cingulate cortex was reported (7). A recent study also reported the decreased functional connectivity pattern homogeneity in lingual gyrus in MDD patients before and after treatment (38). These findings revealed an important role of lingual gyrus in neuropathology of MDD. In addition, we found disrupted functional connectivities between left lingual gyrus with right lingual gyrus, anterior superior temporal gyrus, and dorsal precentral gyrus, and the functional connectivities between left lingual gyrus with right lingual gyrus and right dorsal precentral gyrus were closely associated with disease duration and depression symptoms, respectively. The decreased functional connections found in MDD patients indicated that depression has long-term effects on the visual cognition and somatosensory processing.

We also found decreased LGI in the posterior superior temporal sulcus in MDD patients. The posterior superior temporal sulcus is a multisensory integration area (39) and plays an important role in language processing (40), biological motion (41), facial processing (42, 43). Moreover, the posterior superior sulcus is also involved in social perception and interaction including recognizing, manipulating, and behaving with respect to socially relevant information (44, 45). Thus, the decreased LGI in posterior superior temporal sulcus may suggest the abnormal social emotion processing in MDD patients. Moreover, we found that the LGI in posterior superior temporal sulcus was negatively correlated with the anxiety symptoms, i.e., HAMA scores, which indicated that the posterior superior temporal sulcus may be mainly related to social anxiety.

There are some limitations in this study. First, the samples used in this study are still relatively small, and the findings need to be further validated. Second, this is a cross-sectional study design and the longitudinal MDD study is needed to describe the developmental changes of LGI. Third, the correlation analyses were uncorrected for multiple comparisons due to limited samples.

In conclusion, the present study revealed the decreased LGI in left lingual gyrus and right posterior superior temporal sulcus and disrupted functional connectivities between left lingual gyrus and right lingual gyrus, anterior superior temporal gyrus, and dorsal precentral gyrus. The changed LGI and functional connectivities showed close associations with clinical performances. Our findings suggested that depression has a long-term effect on impaired visual cognitive functions, and the somatosensory symptoms may be an early biomarker for depression remission. Moreover, our findings indicated that posterior superior temporal sulcus may be mainly related social anxiety not emotion processing.

## Data Availability Statement

The raw data supporting the conclusions of this article will be made available by the authors, without undue reservation.

## Ethics Statement

The studies involving human participants were reviewed and approved by the Affiliated Brain Hospital of Guangzhou Medical University Ethics Committee. The patients/participants provided their written informed consent to participate in this study.

## Author Contributions

WK and HW designed the research. HW collected the data. JLo, JX, XW, JLi, and SR analyzed the data and wrote the paper. All authors contributed to the article and approved the submitted version.

## Conflict of Interest

The authors declare that the research was conducted in the absence of any commercial or financial relationships that could be construed as a potential conflict of interest.

## References

[1] Mathers CD Loncar D. Projections of global mortality and burden of disease from 2002 to 2030. PLoS Med. (2006) 3:e442. 10.1371/journal.pmed.0030442 17132052 PMC1664601

[2] Kupfer DJ Frank E Phillips ML. Major depressive disorder: new clinical, neurobiological, and treatment perspectives. Lancet. (2012) 379:1045–55. 10.1016/S0140-6736(11)60602-8 22189047 PMC3397431

[3] Drevets WC Price JL Simpson JR Jr Todd RD Reich T Vannier M. Subgenual prefrontal cortex abnormalities in mood disorders. Nature. (1997) 386:824–7. 10.1038/386824a0 9126739

[4] Drevets WC. Neuroimaging and neuropathological studies of depression: implications for the cognitive-emotional features of mood disorders. Curr Opin Neurobiol. (2001) 11:240–9. 10.1016/S0959-4388(00)00203-8 11301246

[5] Botteron KN Raichle ME Drevets WC Heath AC Todd RD. Volumetric reduction in left subgenual prefrontal cortex in early onset depression. Biol Psychiatry. (2002) 51:342–4. 10.1016/S0006-3223(01)01280-X 11958786

[6] Altshuler L Bookheimer S Proenza MA Townsend J Sabb F Firestine A. Increased amygdala activation during mania: a functional magnetic resonance imaging study. Am J Psychiatry. (2005) 162:1211–3. 10.1176/appi.ajp.162.6.1211 15930074

[7] Wu H Sun H Xu J Wu Y Wang C Xiao J. Changed hub and corresponding functional connectivity of subgenual anterior cingulate cortex in major depressive disorder. Front Neuroanat. (2016) 10:120. 10.3389/fnana.2016.00120 28018183 PMC5159433

[8] Wang C Wu H Chen F Xu J Li H Li H Disrupted functional connectivity patterns of the insula subregions in drug-free major depressive disorder. J Affect Disord. (2017) 234:297–304. 10.1016/j.jad.2017.12.033 29587165

[9] Wu H Sun H Wang C Yu L Li Y Peng H. Abnormalities in the structural covariance of emotion regulation networks in major depressive disorder. J Psychiatr Res. (2017) 84:237–42. 10.1016/j.jpsychires.2016.10.001 27770743

[10] Sun H Luo L Yuan X Zhang L He Y Yao S. Regional homogeneity and functional connectivity patterns in major depressive disorder, cognitive vulnerability to depression and healthy subjects. J Affect Disord. (2018) 235:229–35. 10.1016/j.jad.2018.04.061 29660636

[11] Wang L Wei Q Wang C Xu J Wang K Tian Y. Altered functional connectivity patterns of insular subregions in major depressive disorder after electroconvulsive therapy. Brain Imaging Behav. (2019) 14:753–61. 10.1007/s11682-018-0013-z 30610527

[12] Wang L Yu L Wu F Wu H Wang J. Altered whole brain functional connectivity pattern homogeneity in medication-free major depressive disorder. J Affect Disord. (2019) 253:18–25. 10.1016/j.jad.2019.04.040 31009844

[13] Garver DL Nair TR Christensen JD Holcomb J Ramberg J Kingsbury S. Atrophic and static (neurodevelopmental) schizophrenic psychoses: premorbid functioning, symptoms and neuroleptic response. Neuropsychopharmacology. (1999) 21:82–92. 10.1016/S0893-133X(98)00138-9 10379522

[14] Ansorge MS Hen R Gingrich JA. Neurodevelopmental origins of depressive disorders. Curr Opin Pharmacol. (2007) 7:8–17. 10.1016/j.coph.2006.11.006 17188022

[15] Peterson BS Wang Z Horga G Warner V Rutherford B Klahr KW. Discriminating risk and resilience endophenotypes from lifetime illness effects in familial major depressive disorder. JAMA Psychiatry. (2014) 71:136–48. 10.1001/jamapsychiatry.2013.4048 24369340 PMC3965257

[16] Peng D Shi F Li G Fralick D Shen T Qiu M Surface vulnerability of cerebral cortex to major depressive disorder. PLoS ONE. (2015) 10:e0120704 10.1371/journal.pone.0120704 25793287 PMC4368815

[17] Li G Nie J Wang L Shi F Lin W Gilmore JH. Mapping region-specific longitudinal cortical surface expansion from birth to 2 years of age. Cereb Cortex. (2013) 23:2724–33. 10.1093/cercor/bhs265 22923087 PMC3792744

[18] Li G Wang L Shi F Lyall AE Lin W Gilmore JH. Mapping longitudinal development of local cortical gyrification in infants from birth to 2 years of age. J Neurosci. (2014) 34:4228–38. 10.1523/JNEUROSCI.3976-13.2014 24647943 PMC3960466

[19] Casanova MF Tillquist CR. Encephalization, emergent properties, and psychiatry: a minicolumnar perspective. Neuroscientist. (2008) 14:101–18. 10.1177/1073858407309091 17971507

[20] Pontious A Kowalczyk T Englund C Hevner RF. Role of intermediate progenitor cells in cerebral cortex development. Dev Neurosci. (2008) 30:24–32. 10.1159/000109848 18075251

[21] la Fougere C Grant S Kostikov A Schirrmacher R Gravel P Schipper HM. Where *in-vivo* imaging meets cytoarchitectonics: the relationship between cortical thickness and neuronal density measured with high-resolution [18F]flumazenil-PET. Neuroimage. (2011) 56:951–60. 10.1016/j.neuroimage.2010.11.015 21073964

[22] Petanjek Z Judas M Kostovic I Uylings HB. Lifespan alterations of basal dendritic trees of pyramidal neurons in the human prefrontal cortex: a layer-specific pattern. Cereb Cortex. (2008) 18:915–29. 10.1093/cercor/bhm124 17652464

[23] Petanjek Z Judas M Simic G Rasin MR Uylings HB Rakic P. Extraordinary neoteny of synaptic spines in the human prefrontal cortex. Proc Natl Acad Sci USA. (2011) 108:13281–6. 10.1073/pnas.1105108108 21788513 PMC3156171

[24] Zilles K Palomero-Gallagher N Amunts K. Development of cortical folding during evolution and ontogeny. Trends Neurosci. (2013) 36:275–84. 10.1016/j.tins.2013.01.006 23415112

[25] Zhang Y Yu C Zhou Y Li K Li C Jiang T. Decreased gyrification in major depressive disorder. Neuroreport. (2009) 20:378–80. 10.1097/WNR.0b013e3283249b34 19218876

[26] Han K-M Won E Kang J Kim A Yoon H-K Chang HS. Local gyrification index in patients with major depressive disorder and its association with tryptophan hydroxylase-2 (TPH2) polymorphism. Hum Brain Mapp. (2017) 38:1299–310. 10.1002/hbm.23455 27807918 PMC6866875

[27] Schmitgen MM Depping MS Bach C Wolf ND Kubera KM Vasic N. Aberrant cortical neurodevelopment in major depressive disorder. J Affect Disord. (2019) 243:340–7. 10.1016/j.jad.2018.09.021 30261449

[28] Williams JB. A structured interview guide for the Hamilton depression rating scale. Arch Gen Psychiatry. (1988) 45:742–7. 10.1001/archpsyc.1988.01800320058007 3395203

[29] Hamilton M. The assessment of anxiety states by rating. Br J Med Psychol. (1959) 32:50–5. 10.1111/j.2044-8341.1959.tb00467.x 13638508

[30] Power JD Barnes KA Snyder AZ Schlaggar BL Petersen SE. Spurious but systematic correlations in functional connectivity MRI networks arise from subject motion. Neuroimage. (2012) 59:2142–54. 10.1016/j.neuroimage.2011.10.018 22019881 PMC3254728

[31] Schaer M Cuadra MB Tamarit L Lazeyras F Eliez S Thiran JP. A surface-based approach to quantify local cortical gyrification. IEEE Trans Med Imaging. (2008) 27:161–70. 10.1109/TMI.2007.903576 18334438

[32] Kelly PA Viding E Wallace GL Schaer M De Brito SA Robustelli B. Cortical thickness, surface area, and gyrification abnormalities in children exposed to maltreatment: neural markers of vulnerability? Biol Psychiatry. (2013) 74:845–52. 10.1016/j.biopsych.2013.06.020 23954109

[33] Richman DP Stewart RM Hutchinson JW Caviness VSJr. Mechanical model of brain convolutional development. Science. (1975) 189:18–21. 10.1126/science.1135626 1135626

[34] Essen DCV. A tension-based theory of morphogenesis and compact wiring in the central nervous system. Nature. (1997) 385:313–8. 10.1038/385313a0 9002514

[35] Fujita I. The inferior temporal cortex: architecture, computation, and representation. J Neurocytol. (2002) 31:359–71. 10.1023/A:1024138413082 12815253

[36] Ishai A. Let's face it: it's a cortical network. Neuroimage. (2008) 40:415–9. 10.1016/j.neuroimage.2007.10.040 18063389

[37] Collins JA Olson IR. Beyond the FFA: The role of the ventral anterior temporal lobes in face processing. Neuropsychologia. (2014) 61:65–79. 10.1016/j.neuropsychologia.2014.06.005 24937188 PMC4122611

[38] Wang J Ji Y Li X He Z Wei Q Bai T. Improved and residual functional abnormalities in major depressive disorder after electroconvulsive therapy. Prog Neuropsychopharmacol Biol Psychiatry. (2020) 100:109888. 10.1016/j.pnpbp.2020.109888 32061788

[39] Beauchamp MS Nath AR Pasalar S. fMRI-Guided transcranial magnetic stimulation reveals that the superior temporal sulcus is a cortical locus of the McGurk effect. J Neurosci. (2010) 30:2414–7. 10.1523/JNEUROSCI.4865-09.2010 20164324 PMC2844713

[40] Xiao Y Friederici AD Margulies DS Brauer J. Development of a selective left-hemispheric fronto-temporal network for processing syntactic complexity in language comprehension. Neuropsychologia. (2016) 83:274–82. 10.1016/j.neuropsychologia.2015.09.003 26352468 PMC4780430

[41] Grossman ED Battelli L Pascual-Leone A. Repetitive TMS over posterior STS disrupts perception of biological motion. Vis Res. (2005) 45:2847–53. 10.1016/j.visres.2005.05.027 16039692

[42] Engell AD Haxby JV. Facial expression and gaze-direction in human superior temporal sulcus. Neuropsychologia. (2007) 45:3234–41. 10.1016/j.neuropsychologia.2007.06.022 17707444

[43] Flack TR Andrews TJ Hymers M Al-Mosaiwi M Marsden SP Strachan JWA. Responses in the right posterior superior temporal sulcus show a feature-based response to facial expression. Cortex. (2015) 69:14–23. 10.1016/j.cortex.2015.03.002 25967084

[44] Jack A Morris JP. Neocerebellar contributions to social perception in adolescents with autism spectrum disorder. Dev Cogn Neurosci. (2014) 10:77–92. 10.1016/j.dcn.2014.08.001 25170555 PMC6987881

[45] Lee SM Gao T McCarthy G. Attributing intentions to random motion engages the posterior superior temporal sulcus. Soc Cogn Affect Neurosci. (2014) 9:81–7. 10.1093/scan/nss110 22983598 PMC3871733

